# Fabrication Process and Surface Morphology Prediction of Radial Straight Groove-Structured CBN Grinding Wheel by Laser Cladding

**DOI:** 10.3390/ma18204733

**Published:** 2025-10-15

**Authors:** Zhelun Ma, Wei Zhang, Qi Liu, Liaoyuan Chen, Chao Zhang, Changsheng Liu, Tianbiao Yu, Qinghua Wang

**Affiliations:** 1 School of Mechanical Engineering and Automation, Northeastern University, Shenyang 110819, China; 2 Key Laboratory of High-End Equipment Intelligent Design and Manufacturing Technology, School of Mechanical Engineering and Automation, Northeastern University, Shenyang 110819, China; 3 Key Laboratory for Anisotropy and Texture of Materials, Ministry of Education, School of Materials Science and Engineering, Northeastern University, Shenyang 110819, China; csliu@mail.neu.edu.cn; 4 Foshan Graduate School of Innovation, Northeastern University, Foshan 528312, China; 5 School of Mechatronics Engineering, Henan University of Science and Technology, Luoyang 471023, China; lychen8790@haust.edu.cn; 6 School of Mechanical Engineering, Southeast University, Nanjing 211189, China

**Keywords:** structured grinding wheel, surface morphology prediction, surface roughness, grinding mechanism

## Abstract

Structured CBN (cubic boron nitride) grinding wheels usually have a specially designed texture on their surface to reduce the grinding heat and grinding force. However, most structured grinding wheels are fabricated by electroplating, brazing, sintering, and mechanical or laser removal on the surface of conventional grinding wheels, which may have problems such as complicated processes, low processing efficiency, and unstable effects. In this paper, additive manufacturing was used to fabricate a radial straight groove-structured grinding wheel. Meanwhile, a corresponding mathematical model of the grinding wheel was also established considering the shape and position of the abrasive grains. Subsequently, the ground surface morphologies of the fabricated wheel and simulated wheel under different machining parameter conditions were compared to further prove the rationality of the simulated grinding wheel. The results showed that the ground surfaces of the fabricated wheel and simulated wheel had similar morphological characteristics. The trend in the surface roughness under the different machining parameter conditions was also analyzed and showed the same variation for fabricated and simulated wheels; the error rate was confined within 8%. This paper elucidates the grinding mechanism and surface morphology formation process of a radial straight groove-structured grinding wheel fabricated by additive manufacturing.

## 1. Introduction

The structured grinding wheel is a novel type of wheel whose working surface is endowed with three-dimensional features such as grooves, blind holes, or protrusions by manufacturing processes. It has played a vital role in the field of precision manufacturing in the past decade [1,2]. The key geometric descriptors governing the wheel morphology are the groove width, blind hole/bump diameter, depth, spacing, orientation angle, overall structuring ratio (fraction of structured area), and so on. Based on the characteristic dimensions of the wheel morphology, structured grinding wheels are classified into macro-structured and micro-structured variants [3,4]. Structured grinding wheels can significantly enhance the fluid-dynamic characteristics of the cutting zone and substantially attenuate thermal loading by precisely controlling the spatial distribution of abrasive grains. Consequently, they exhibit unique advantages in mitigating wheel clogging, grinding burn, and other critical challenges in precision machining [5,6,7].

In the past decade, many researchers have focused on the fabrication of structured grinding wheels. Structured grinding wheels are prepared by electroplating, brazing, sintering, and mechanical or laser removal on the surface of conventional grinding wheels [8,9,10]. Deng et al. [11] utilized a laser to fabricate a structured grinding wheel with hydrophilia. The scanning times and focus position were synergistically controlled to fabricate a structured grinding wheel with regular hexagonal structures. Yu et al. [12,13] fabricated a structured grinding wheel based on combinatorial bionics and optimized its hydrodynamic properties. These studies showed that a fish scale-structured grinding wheel demonstrates markedly superior performance in fluid dynamics. Yi et al. [14] studied the grinding force of a straight groove-structured grinding wheel and established the grinding force model. The results showed that a greater intermittent ratio of the structured grinding wheel has a more significant effect on weakening the grinding force. Guo et al. [15] established a model of a micro-structured micro-grinding tool, optimized the fabrication process, and analyzed the grinding performance. Moreover, Wu and Guo et al. [16,17] also fabricated a structured grinding wheel via laser ablation technology, in which the textures were smaller than the abrasive grains, and analyzed the grinding performance of a silicon carbide workpiece and quartz glass. Yang et al. [18] investigated the grinding force based on a grinding wheel with an ordered arrangement of abrasive grains. Zhang et al. [19] fabricated a structured grinding wheel via monolayer technology and studied the effect of texture parameters on grinding performance. However, these structured grinding wheels were all fabricated by electroplating, brazing, sintering, and mechanical or laser removal on the surface of conventional grinding wheels, which have problems such as complicated processes, low processing efficiency, and unstable effects.

Conventional structured grinding wheels are commonly manufactured via a subtractive manufacturing method, in which controlled material removal from the abrasive layer is performed to create the desired macro- or micro-scale features [20,21]. Now, the development of the additive manufacturing (AM) technique and advanced powder technique [22] provides a paradigm shift for structured grinding wheel fabrication, enabling material to be built up layer by layer by data-driven methods. In the matrix-bonding-abrasive system, AM concurrently integrates macro-scale coolant channels, micro-scale textures, and hierarchical pore networks. Li and Wan et al. [23,24,25] fabricated a metal-bonded grinding wheel with a specific porous structure and high porosity by additive manufacturing and proved the good performance of this structured grinding wheel via an experiment grinding BK7 glass. Barmouz et al. [26] fabricated a resin-bonded grinding wheel by additive manufacturing, which can achieve internal cooling along with surface structures and enhance the performance and life of the grinding wheel. Yang et al. [27] used additive manufacturing technology to fabricate a diamond grinding wheel with a regular grain distribution. Li and Wei et al. [28] proposed a hollow ceramic grinding wheel by 3D printing, which was applied in biological bone grinding. These studies all indicate that the grinding wheels fabricated by additive manufacturing have better grinding performance or are applied in a special field. However, the grinding process simulation of laser-cladding structured CBN grinding wheels has rarely been reported. The grinding mechanism is still not clear. Therefore, it is very necessary to analyze the grinding process of radial straight groove-structured CBN grinding wheels through mathematical models.

In this paper, a mathematical model of a radial straight groove-structured grinding wheel was established. The shape and position of the abrasive grains were discussed, and the surface morphology of this structured grinding wheel was simulated. Subsequently, the radial straight groove-structured grinding wheel was fabricated by additive manufacturing. Comparisons were made between the fabricated grinding wheel and the simulated grinding wheel to evaluate the reliability and rationality of the simulated grinding wheel. Finally, the ground surface morphologies of the fabricated grinding wheel and the simulated grinding wheel were also compared, and the surface roughness was analyzed to further evaluate the grinding performance. This work can offer significant guidance for the preparation and use of structured grinding wheels in additive manufacturing.

## 2. Modelling

### 2.1. Grinding Wheel Modeling

#### 2.1.1. CBN Abrasive Grain Model

**Figure 1:**
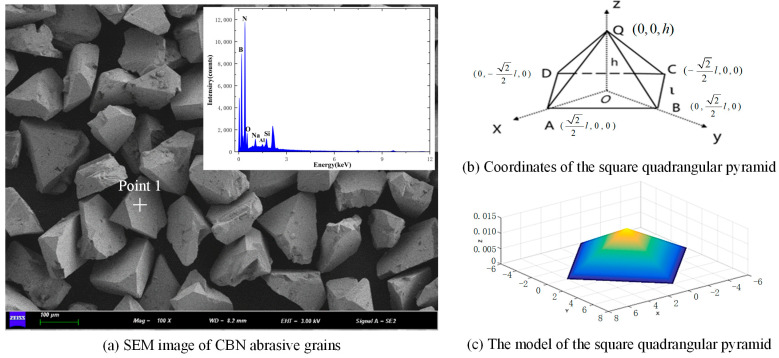
CBN abrasive grain model.

Due to the stochastic nature of CBN (cubic boron nitride) abrasives, CBN abrasive grain modeling is quite challenging. As shown in Figure 1a, the CBN abrasives are mostly polyhedral with edges. Therefore, to facilitate the grinding mechanism, this study starts with a simplified model. Considering the micro-structuring (the abrasive grain tips are always exposed during grinding) and the geometric shape of CBN abrasives, a square-based pyramid model is chosen for the abrasive grains. Figure 1b,c show the coordinates and model of the CBN abrasive grain model.

**Figure 2:**
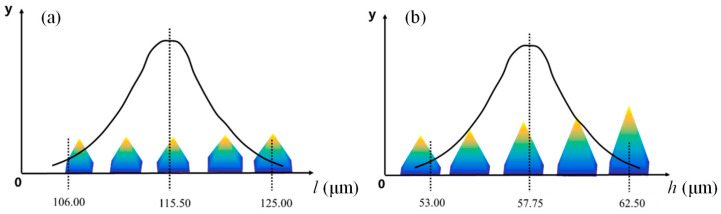
The size distribution of CBN abrasive grains: (**a**) the size range of *l*; (**b**) the size range of *h*.

Once the abrasive grain shape is fixed, its size must also be determined. In the conventional grinding process, the grain size is often characterized by mesh size. The CBN abrasives used in this paper have a mesh size of 120–140. To align the size of the abrasive grain model with the actual size, the base edge length *l* of the square-based pyramid model is set in the range of 106–125 μm. According to previous studies, CBN abrasives can protrude at a height of 50% of their diameter on the laser-cladding layer. Therefore, the height *h* of the square-based pyramid model is set between 53 and 62.5 μm. The base edge length *l* and height *h* both follow a normal distribution, i.e., *l*~*N*(115.50, 3.1670^2^), *h*~*N*(57375, 1.5835^2^), which are shown in Figure 2.

**Figure 3:**
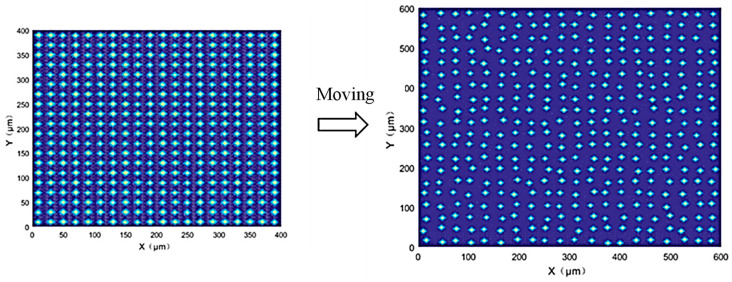
A schematic diagram of the random movement of abrasive grains.

The spatial distribution of abrasive grains is also crucial for simulating the grinding process. Some researchers assume that the abrasive grains are uniformly spaced [29]. During the actual process, it is difficult to ensure a uniform distribution of abrasive grains in a grinding wheel. To make the simulated grinding wheel surface better match the actual one, a random spatial distribution of abrasive grains in the grinding wheel must be considered in the simulation. In this paper, two random movement variables (*x*-axis and *y*-axis) are added to the center coordinates of the abrasive grains, thereby causing position variation of the abrasive grains on the horizontal plane. Figure 3 shows a schematic diagram of the random movement of abrasive grains.

#### 2.1.2. Grinding Wheel Morphology

**Figure 4:**
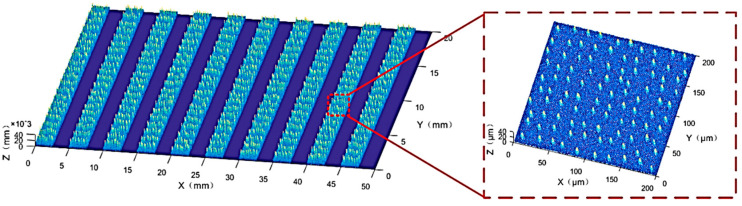
A circumferential drawing and a local magnification drawing of the grinding wheel morphology model.

It is well known that the actual surface topography of a grinding wheel is extremely complex. However, if it is assumed that only the surface-layer abrasive grains participate in grinding, a single-abrasive-grain-layer grinding wheel model could be established without considering the abrasive grain wear. From the macro-structural perspective, the structured grinding wheel offers more chip removal space, which can effectively prevent wheel blockage. From the micro-structural perspective, the micro-arrangement of abrasive grains in the wheel provides more retention space for hard-to-machine materials prone to adhesion, thus effectively alleviating abrasive grain shedding caused by adhesion. Therefore, the structured grinding wheel designed in this paper features abrasive grains only in specific areas, namely the abrasive regions, while the rest are non-abrasive regions. These two types of regions, in a 1:1 ratio, are arranged alternately. This turns the grinding process into an intermittent one, offering more space and time for chip removal. Figure 4 shows a circumferential drawing and a local magnification drawing of the grinding wheel morphology model.

### 2.2. Simulation of Ground Surface Morphology

The essence of grinding lies in the relative motion between abrasive grains and the workpiece material, which removes the surface material of the workpiece and forms the ground surface. The uncertainty of internal grinding wheel factors and external factors makes it extremely difficult to predict the topography of the ground surface. Internal factors mainly involve the complexity of the grinding wheel surface, including the irregular abrasive grain shape, random protrusion heights and positions, and grain wear and deformation. External factors include the grinding wheel dynamic balance, machine tool spindle runout, and workpiece material properties. These factors make it hard to predict the cutting-edge motion trajectory during grinding. Therefore, the following assumptions are made in this study:

(1) Abrasive grain deformation and wear are neglected, and grains are considered rigid bodies during the grinding process.
(2) Workpiece material deformation is ignored; material in contact with grains is directly removed.
(3) The vibrations of the machine and grinding wheel during grinding are not considered.

**Figure 5:**
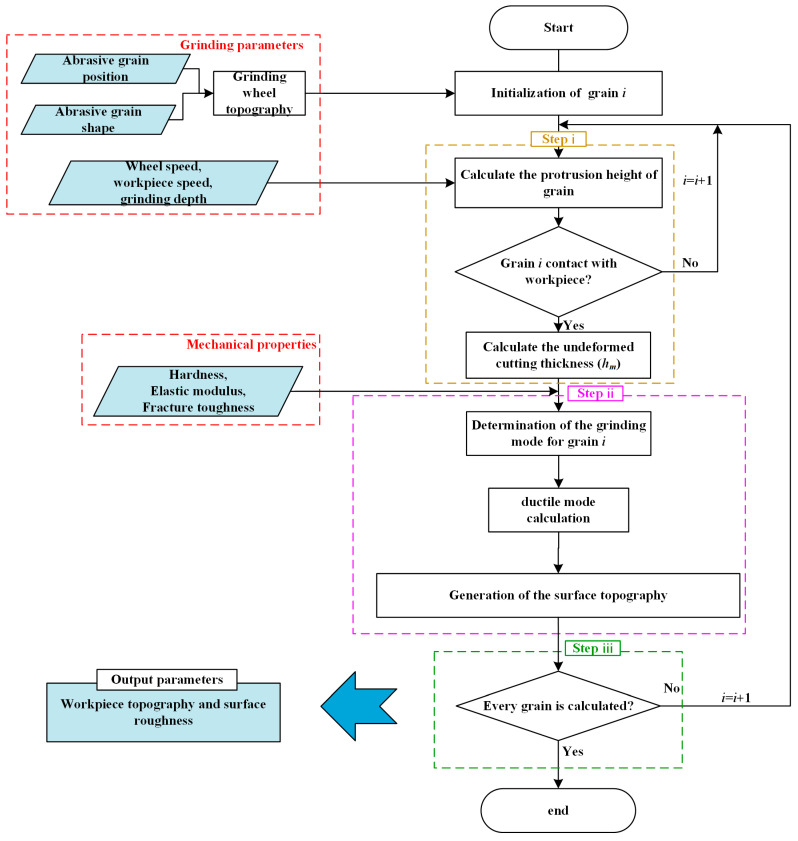
Framework of the simulated analytical model.

The entire framework for the ground surface morphology predictive model is shown in Figure 5. The grinding parameters and mechanical parameters could be systematically added to the calculation process; thus, the surface morphology would be predicted by the kinematics equations of the abrasive grains.

#### 2.2.1. Motion Trajectory of Abrasive Grain

**Figure 6:**
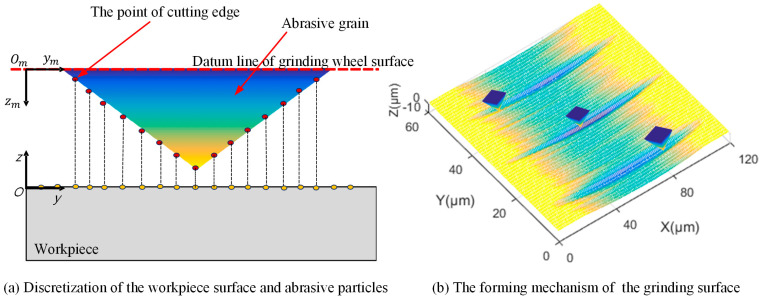
The grinding mechanism of a single abrasive grain.

During the grinding process, the movement trajectory of each abrasive grain, where it interferes with the workpiece material, directly determines the ground surface morphology. Thus, analyzing these trajectories is key to predicting the surface formation. To conduct the simulations, both the workpiece surface and abrasive grains need discretization. As shown in Figure 6a, in the workpiece coordinate system *OYZ*, the ground surface is represented by a topological matrix *S*, where *S*(i, j) denotes the element in the *i*th row and *j*th column. The initial workpiece surface is assumed to be flat, so the initial value of *S* is set to 0. Accordingly, to reduce the computational load, each abrasive grain is simplified from 3D to 2D before discretization. This is because the maximum grain profile perpendicular to the feed direction determines the workpiece surface groove shape. The discretized abrasive grains can be regarded as a series of point-cutting edges, which serve as the basic units for simulation operations.

#### 2.2.2. Model of Surface Morphology Generation

The motion trajectory of the grinding process for the square-based pyramid abrasive grain model is derived. Using the cutting edge of the abrasive grain as the smallest calculation unit, the motion trajectory of the *j*th cutting edge from time *t*_0_ to time *t*_0_ + *kΔt* can be expressed as follows [30]:

(1) $$\left\{\begin{array}{l} x=x_{0}+v_{s}\times k\Delta t\\ z=\frac{x^{2}}{\left(d_{s}+2h_{j}\right){\left(1+\frac{v_{w}}{v_{s}}\right)}^{2}}-h_{j} \end{array}\right.$$

where *d_s_* is the diameter of the grinding wheel; *v_w_* is the feeding speed; *v_s_* is the grinding wheel speed; *h_j_* is the protrusion height of the *j*th cutting edge; and *x*_0_ is the initial abscissa value of the cutting edge. The aggregation of multiple cutting edges’ trajectories forms the ground surface morphology on the workpiece surface (in Figure 6b).

To describe the motion of all abrasive grains during the grinding process, a global coordinate system is defined. The initial workpiece plane coincides with the nominal surface of the grinding wheel at the z-axis origin during grinding initialization, and the leftmost end of the workpiece surface serves as the x-axis origin. Due to the stochasticity of grain size, assuming a total of *n* abrasive grains, each with a varying number of cutting edges *m*_(*g*)_, the trajectories of multiple grains can be represented by the mathematical set *P* as shown in Equation (2).

(2) $$\left\{\begin{array}{l} x^{\left(i\right)}={x_{0}}^{\left(i\right)}+v_{s}\times k\Delta t\\ z^{\left(i\right)}=\frac{{\left(x^{\left(i\right)}\right)}^{2}}{\left(d_{s}+2{h_{j}}^{\left(i\right)}\right){\left(1+\frac{v_{w}}{v_{s}}\right)}^{2}}-{h_{j}}^{\left(i\right)} \end{array}\right.\begin{array}{l} \begin{array}{l} \left(j=1,2,\cdots ,\;m_{\left(g\right)}\right)\\ \left(g=1,2,\cdots ,n\right) \end{array} \end{array},\begin{array}{l} \left(i=1,2,\cdots ,n\right) \end{array}$$

Here, *i* is the number associated with a particular abrasive grain. Additionally, the ground surface morphology is formed by the contours of different abrasive grains engaging with the workpiece. Thus, it requires iterative updates following each grain’s interaction. Based on the assumptions of this paper, the groove profiles generated by different grains are independent of each other. Consequently, the final ground surface is formed by the lowest points of all these groove profiles, which is shown in Equation (3).

(3) $$z=\min\left(z^{\left(1\right)},z^{\left(2\right)},z^{\left(3\right)},\cdots ,z^{\left(n\right)},0\right)$$

## 3. Experiment Method

### 3.1. Fabrication of Laser-Melting Structured CBN Grinding Wheel

**Figure 7:**
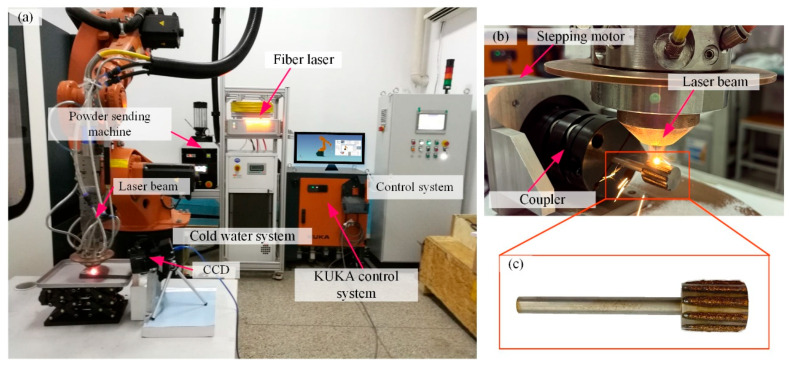
Experimental set-up of grinding wheel fabrication: (**a**) laser melting system; (**b**) process of fabricating laser-melting structured CBN grinding wheel; (**c**) structured CBN grinding wheel.

The laser cladding system consists of a 6-degree-of-freedom KUKA robot and fiber laser (IPG Company, New York, NY, USA). The whole control system is designed by Yuchen Company (Nanjing, China), including the powder feeder and so on. Figure 7 shows a schematic diagram and the experimental set-up of this experiment.

**Figure 8:**
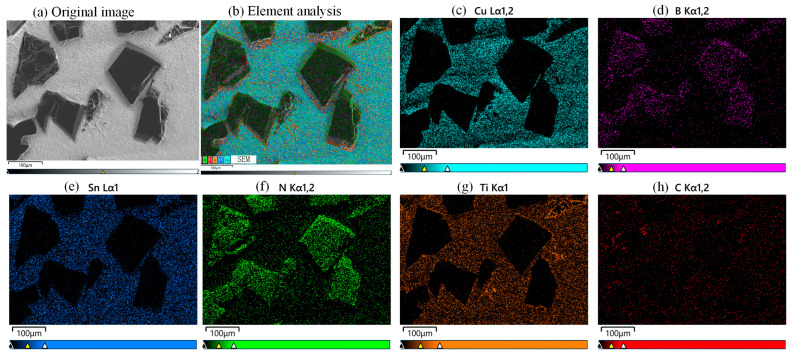
Element surface scanning analysis of the CBN grinding wheel surface morphology.

The grinding wheel matrix is made of Hardened 45 steel. The bond is a Cu-Sn-Ti powder with a mesh size of 100-250. The abrasive grains are CBN-980 T (Funik Ultrahard Material Co., Ltd., Zhengzhou, China) with a mesh size of 120–140. There is a significant difference in melting points between the bond and the matrix, with the matrix having a higher melting point. Excessive temperatures would damage the CBN abrasive grains, so a two-layer printing method is used. First, a bond material layer is printed onto the matrix, which is called the bond layer. Then, a layer of grain–bond material mixture is printed onto this bond layer, which is called the grain layer. This enables the second layer to form a melt pool and bond with the first layer at a lower temperature, preventing the abrasive grains from burning out. The machining parameters of the grinding wheel fabrication can be found in Table 1. Figure 8 shows the element surface scanning analysis of the CBN grinding wheel surface morphology, which can prove the authenticity of the materials.

When the grinding wheel is being prepared, the laser generator operates intermittently. The KUKA robot moves the laser beam along the wheel surface in a straight line. A stepper motor, connected to the wheel matrix via a coupler, is also controlled to rotate intermittently. When the laser completes a straight-line path, the motor rotates the matrix by a set angle. For instance, to fabricate 10 cladding layers, the wheel rotates 36° each time, with the laser making a straight-line movement, until the wheel completes a full rotation. Figure 7c shows the finished grinding wheel. The prepared grinding wheel needs diamond-roller dressing and sharpening to ensure cylindricity within 2 μm.

### 3.2. Grinding Experiment

**Figure 9:**
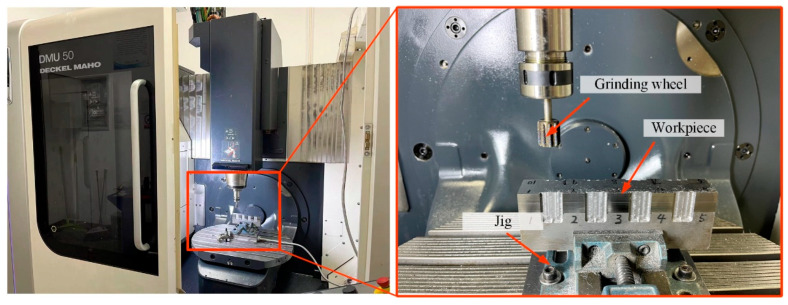
The grinding process in this paper.

To further evaluate the grinding performance of the laser-melting structured CBN grinding wheel, a series of grinding experiments was carried out. Figure 9 shows the equipment for the grinding experiments. A machining center (DMG U50, Bielefeld, Germany) was utilized to implement the whole experiment. The mechanical properties of the workpiece can be found in Table 2, and the machining parameters of the grinding experiment can be found in Table 3.

Furthermore, a 3D laser scanning confocal microscope (OLYMPUS LEXT OLS4100, Tokyo, Japan) was utilized to measure the surface morphology and surface roughness. The bundled software (OLS4100) was used to analyze the surface morphology and surface roughness. All other relevant settings were set according to the manual. On the basis of the software manual, the cutoff length was set to 800 μm, and all other relevant settings were configured according to the manual’s standards. Ten different ground surfaces were selected to measure the surface roughness to ensure the reliability of the results.

## 4. Discussion and Analysis

### 4.1. Comparison of Grinding Wheel Models

**Figure 10:**
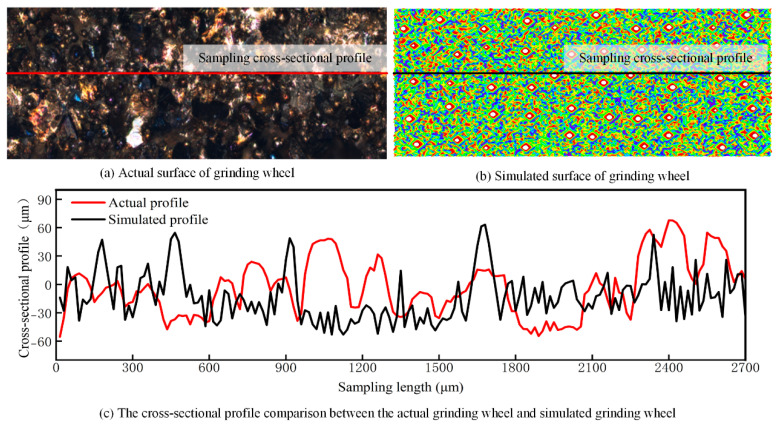
A comparison between the fabricated grinding wheel and the simulated grinding wheel.

Figure 10 shows a comparison between the fabricated grinding wheel and the simulated grinding wheel. It can be observed that although the two cross-sectional profiles have different peak–valley pairs, their overall fluctuation ranges are essentially the same, with an error rate of less than 5%. Therefore, the simulated grinding wheel model closely matches the real one.

### 4.2. Analysis of Grinding Surface Morphology

#### 4.2.1. Wheel Speed

**Figure 11:**
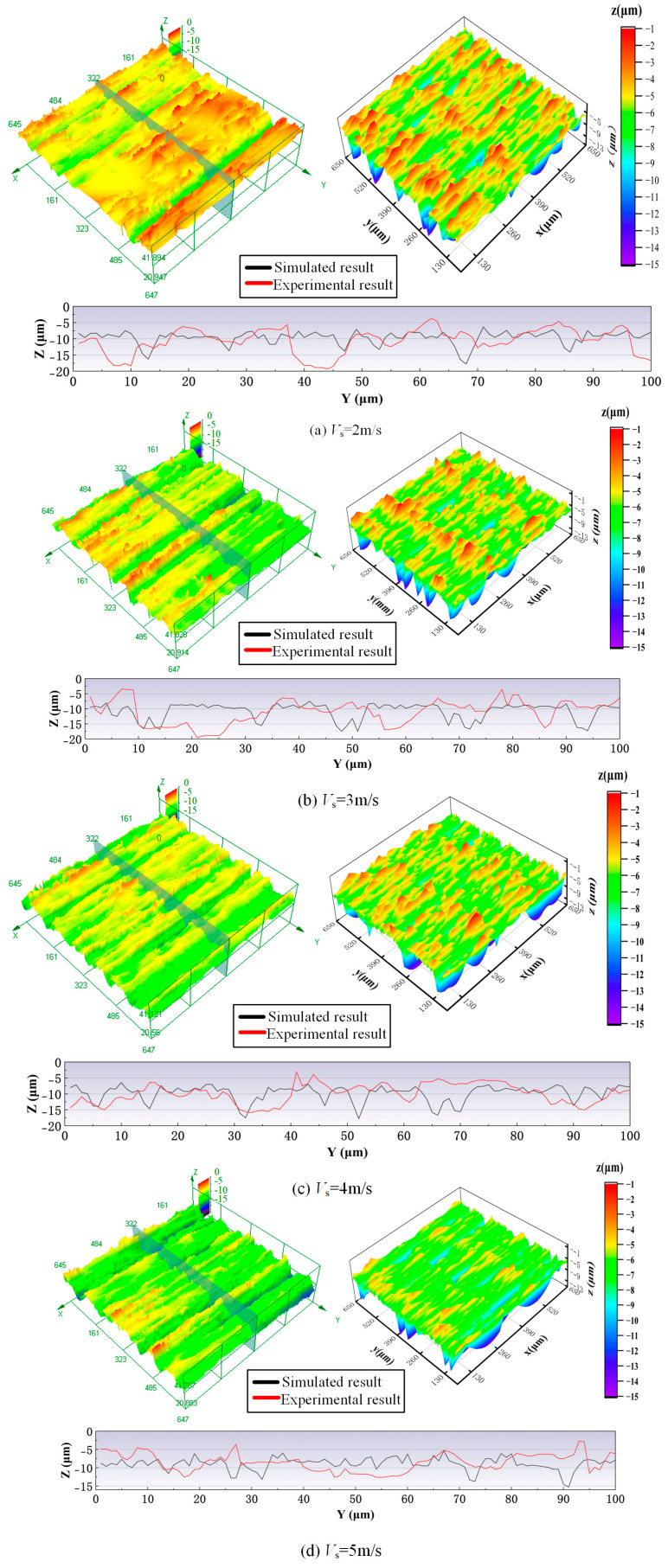
A comparison of experimental and simulated surface morphologies of the workpiece under different grinding wheel speed conditions: feeding speed 2 mm/s, grinding depth 15 μm.

Figure 11 shows a comparison of experimental and simulated surface morphologies of the workpiece under different grinding wheel speed conditions. As the grinding wheel speed increases, the distance between the peaks along the grinding direction on the machined surface decreases, the surface waves become more closely spaced, the overall surface becomes smoother, and the average surface height is reduced. This is because as the grinding wheel speed increases, the distance that the workpiece travels during one revolution of the wheel decreases, resulting in more closely spaced surface waves. Additionally, since the duration of each cutting cycle is reduced, the intersection coordinates between adjacent cutting cycles also decrease, leading to a reduction in the overall height of the ground surface. As shown in Figure 11, the simulated results are in great agreement with the experimental results. At the same scale, they have similar morphological features. When the wheel speed increases from 2 m/s to 5 m/s, the surface becomes smoother. To further analyze the similarity between the simulated and experimental results, a cross-sectional profile with a length of 100 μm was compared. Although the simulated and experimental profiles exhibit different peaks and valleys, their overall fluctuation ranges are essentially the same.

#### 4.2.2. Feeding Speed

**Figure 12:**
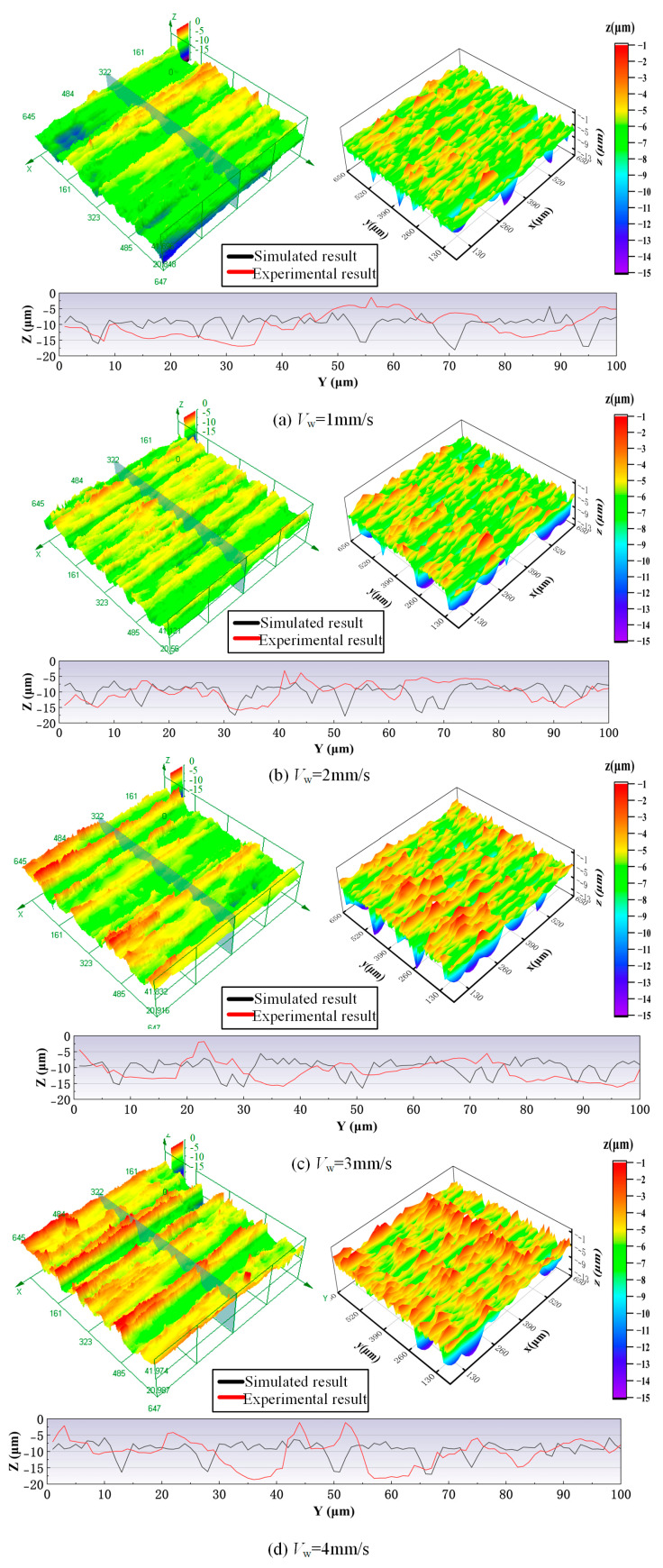
A comparison of experimental and simulated surface morphologies of the workpiece under different feeding speed conditions: grinding wheel speed 3 m/s, grinding depth 15 μm.

Figure 12 shows a comparison of experimental and simulated surface morphologies of the workpiece under different feeding speed conditions. As the feeding speed increases, the surface quality of the workpiece declines. More effective abrasive grains boost the material removal rate and machining efficiency. However, the undeformed chip thickness per grain also increases, which causes a significant rise in the height of surface peaks and valleys and a more scattered profile distribution. Therefore, the regularity of the surface texture weakens, and the non-uniformity of the groove distribution intensifies. When the feeding speed increases from 1 mm/s to 4 mm/s, the surfaces become rougher. The reason for this is that increasing the feeding speed shortens the spacing of abrasive grain cutting trajectories, promoting the energy density and intensifying local surface distortion.

#### 4.2.3. Grinding Depth

**Figure 13:**
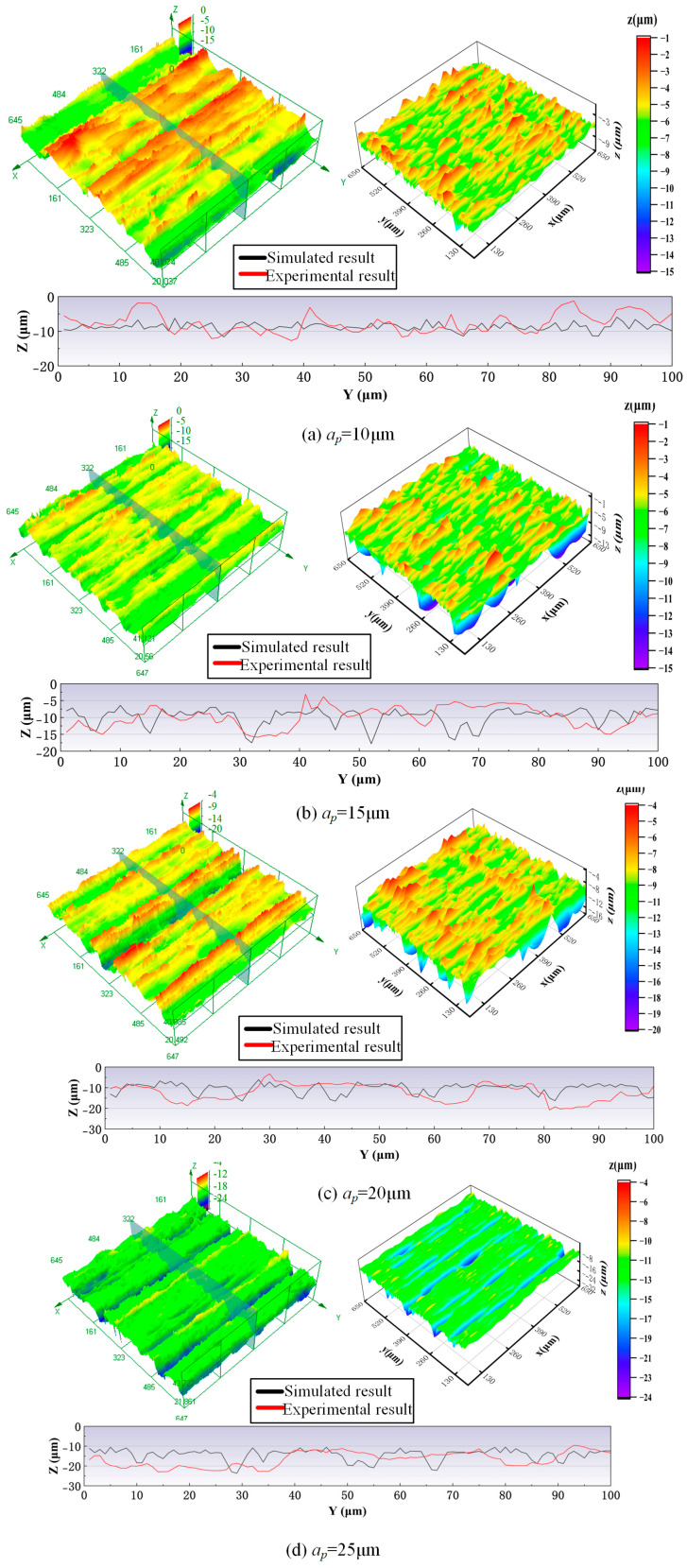
A comparison of experimental and simulated surface morphologies of the workpiece under different grinding depth conditions: grinding wheel speed 3 m/s, feeding speed 2 mm/s.

Figure 13 shows a comparison of experimental and simulated surface morphologies of the workpiece under different grinding depth conditions. When the grinding depth is 10 μm, the profile height of the workpiece surface is distributed centrally with a regular feed-direction texture. The experiments and simulations both show a relatively flat surface morphology. This is because the small grinding depth leads to fewer high-protruding grains participating in the cutting process due to grain height differences and their random distribution. The smaller contact area results in a lower chip thickness per grain and less material removal, thus maintaining high surface flatness. As the grinding depth continues to increase, more abrasive grains participate in the cutting process. The material removal efficiency is improved, and the differences in cutting depth among grains become more pronounced. This leads to increased dispersion in the peak–valley distribution of the surface morphology, reduced regularity in texture, and a more complex overall surface morphology. The theoretical analysis proves that when the grinding depth exceeds the threshold value, the spatial distribution of cutting trajectories no longer changes, and the surface morphology stabilizes. However, during the actual grinding process, increased grinding depth can significantly change the cutting dynamics. The greater undeformed chip thickness per abrasive grain intensifies the grinding force fluctuations and causes plowing and rubbing effects. These phenomena increase surface plastic deformation and large-scale brittle fracture. Furthermore, the higher cutting load accelerates abrasive grain wear and failure (e.g., breakdown and grain pull-out), further degrading surface quality.

Furthermore, the comparison of cross-sectional profiles reveals that the actual ground surface has greater fluctuations along the wheel-axial direction in experiments than in simulations. This discrepancy stems from structural characteristic differences between the simulation model and real grinding wheels. In simulations, the abrasive grains are evenly distributed on the wheel’s outer-circular reference surface, with protrusion height differences alone modeling variation. However, actual grinding wheels show significant surface-grain distribution heterogeneity due to dressing process deviations, grinding-induced surface damage, and local wear from workpiece irregularities. This creates macro-scale topographic features. In summary, despite these differences, the simulation model still effectively captures the core features of the actual grinding surface morphology.

### 4.3. Analysis of Surface Roughness

**Figure 14:**
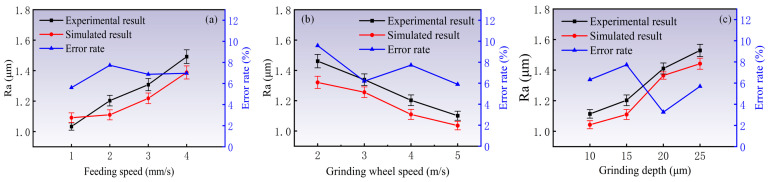
Simulated and experimental results of the surface roughness under various grinding wheel speeds, feeding speeds, and grinding depths: (**a**) grinding wheel speed; (**b**) feeding speed; (**c**) grinding depth.

To further verify the reliability and rationality of the simulation in this paper, the surface roughness was analyzed. Therefore, the Ra parameter of surface roughness was utilized to evaluate the surface quality [32]. Figure 14 shows the simulated and experimental results of the surface roughness under various grinding wheel speeds, feeding speeds, and grinding depths. As shown in Figure 14a, as the grinding wheel speed increases, Ra value decreases monotonically. When the wheel speed increases from 2 m/s to 5 m/s, the surface *Ra* value decreases from 1.461 μm to 1.101 μm, which represents a reduction of 24.61%. The experimental and simulated results exhibit consistent overall trends. Their maximum error rate is only 8.3%. As the grinding wheel speed increases (see Figure 14b), the *Ra* value increases accordingly. When the feeding speed increases from 1 mm/s to 4 mm/s, the surface *Ra* value increases from 1.033 μm to 1.492 μm, which represents an increase of 44.43%. The error rate is confined within 7.7%. As the grinding depth increases (see Figure 14c), the *Ra* value also increases accordingly. When the wheel speed increases from 10 μm to 25 μm, the surface *Ra* value decreases from 1.114 μm to 1.529 μm, which represents an increase of 37.25%. The error rate is confined within 8%.

In summary, an increase in wheel speed has a positive effect on workpiece surface roughness, whereas a higher feeding speed and greater grinding depth exert a detrimental effect. Increasing the wheel speed reduces the undeformed chip thickness, whereas increasing the feeding speed or grinding depth enlarges it. Both the surface roughness and surface morphology are intimately related to the undeformed chip thickness: when it increases, the larger extrusion force from the abrasive grains drives more material laterally, forming deeper grooves and increasing the propensity for chip adhesion.

The experimental surface roughness values have the same trend as the simulated ones across all grinding parameters, yet they are systematically higher than the predictions. This discrepancy is attributable to inherent systematic errors in the simulation model, which considers only idealized grain–workpiece pure cutting while neglecting additional phenomena that influence the real surface morphology. During the actual grinding process, besides the grinding wheel morphology and mechanical vibrations, factors such as grinding forces, elevated temperatures, material pile-up, and side flow caused by plastic deformation, as well as chip adhesion and molten droplets, all contribute to surface roughness. These unmodeled effects become more pronounced as machining parameters vary, thereby amplifying the divergence between simulations and measurements. Nevertheless, the overall influence of the grinding parameters on surface roughness remains consistent.

## 5. Conclusions

In this paper, a mathematical model of a radial straight groove-structured grinding wheel was established. The shape and position of the abrasive grains were discussed, and the surface morphology of this structured grinding wheel was simulated. Subsequently, the radial straight groove-structured grinding wheel was fabricated by additive manufacturing. Comparisons were made between the fabricated grinding wheel and the simulated grinding wheel to evaluate the reliability and rationality of the simulated grinding wheel. Finally, the ground surface morphologies of the fabricated grinding wheel and the simulated grinding wheel were also compared, and the surface roughness was analyzed to further evaluate the grinding performance. The main conclusions of this paper are as follows:

(1) Additive manufacturing technology was used to fabricate a radial straight groove-structured grinding wheel. Meanwhile, a corresponding mathematical model of the grinding wheel was also established considering the shape and position of the abrasive grains. A comparison of the wheel surface morphology substantiated the reliability and rationality of the simulated grinding wheel. The results showed that although the wheel surface morphology had different peak–valley pairs, their overall fluctuation ranges were essentially the same, with an error rate of less than 5%. Therefore, the simulated grinding wheel model closely matched the real one.
(2) The ground surface morphologies of the fabricated wheel and simulated wheel under different machining parameter conditions were compared to further prove the rationality of the simulated grinding wheel. The results showed that the ground surfaces of the fabricated wheel and simulated wheel had similar morphological characteristics, which proved the correctness of the grinding mechanism analysis for the radial straight groove-structured grinding wheel.
(3) The trend in the surface roughness under different machining parameter conditions was also analyzed and showed the same variation for the fabricated and simulated wheels; the error rate was confined within 8%. The discrepancy is attributable to inherent systematic errors in the simulation model, which considers only idealized grain–workpiece pure cutting while neglecting additional phenomena that influence the real surface morphology.

## Figures and Tables

**Table 1:** Machining parameters of grinding wheel fabrication.

Parameters	Value	
	Bond Layer	Grain Layer
Laser power (W)	400	360
Powder feeding speed (r/min)	1.3	1.6
Powder feed gas rate (L/min)	8	8
Shielding gas rate (L/min)	15	15
Scanning speed (mm/s)	6.5	5

**Table 2:** The mechanical properties of the workpiece [31].

Mechanical Property	Value
Density (g/cm^3^)	7850
Poisson’s ratio	0.269
Elasticity modulus (Gpa)	210
Hardness (HRC)	48
Yield strength (MPa)	205

**Table 3:** The machining parameters of the grinding experiment.

Parameters	Value
Grinding condition	Up-grinding (without cooling liquid)
The size of the workpiece (mm)	180 × 60 × 30
Wheel speed (m/s)	2, 3, 4, 5
Feeding speed (mm/s)	1, 2, 3, 4
Grinding depth (μm)	10, 15, 20, 25

## Data Availability

The original contributions presented in this study are included in the article. Further inquiries can be directed to the corresponding authors.
